# Bromochloroacetonitrile Cytotoxicity and Regenerative Responses in HaCaT and HUVEC Cells

**DOI:** 10.1021/acsomega.6c02428

**Published:** 2026-04-29

**Authors:** Elif Fayadoglu, Mustafa Fayadoglu, Banu Barutca, A. Tansu Koparal, A. Savas Koparal

**Affiliations:** 1 Institute of Graduate Programs Department of Biology, Programme of Molecular Biology, Eskişehir Technical University, Tepebaşı, Eskişehir 26470, Turkey; 2 Biotechnology Institute, Ankara University, Ankara 06135, Turkey; 3 Yunus Emre Vocational School of Health Services, Department of Medical Services and Techniques, Anadolu University, Eskişehir 26470, Turkey

## Abstract

Bromochloroacetonitrile (BCAN) is a halogenated nitrogenous disinfection byproduct (N-DBP) frequently detected in chlorinated swimming pools. While its cytotoxicity has been established in various mammalian cells, its dermal and vascular impacts remain poorly understood. This study evaluated the cytotoxic, oxidative, and regenerative responses of human keratinocytes (HaCaT) and vascular endothelial cells (HUVECs) to BCAN exposure in vitro. BCAN significantly reduced HaCaT viability in a concentration- and time-dependent manner, with an IC_50_ of ∼42 μM at 48 h. Increased lactate dehydrogenase (LDH) release and reactive oxygen species (ROS) generation confirmed membrane damage and oxidative stress. In contrast, HUVECs maintained viability and membrane integrity at all concentrations except 80 μM, indicating higher cellular resilience. Representative scratch and tube formation assays suggested that keratinocyte migration and endothelial angiogenic behavior were largely preserved, with only moderate impairment at cytotoxic concentrations. These findings highlight the selective sensitivity of skin cells to BCAN and suggest that short-term BCAN exposure may have limited effects on wound closure- and angiogenesis-related responses under the present experimental conditions. The results underscore the importance of evaluating regenerative end points in the risk assessment of unregulated DBPs in recreational waters.

## Graphical abstract

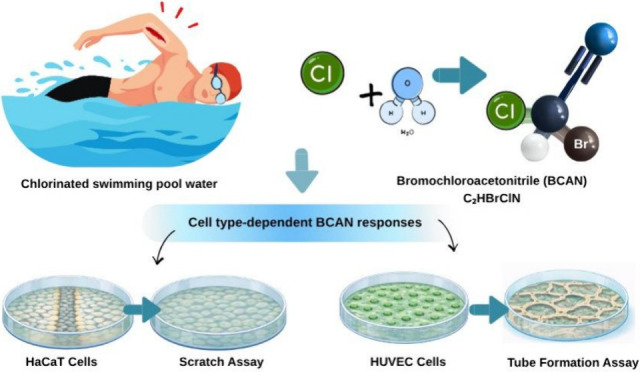

## 1 Introduction

Swimming is a common form of exercise embraced by individuals of various ages and socioeconomic groups, primarily because of its recognized positive effects on health.^1–3^ Nevertheless, these advantages can only be sustained if swimming pool environments are managed with appropriate hygienic practices to minimize potential health risks. During pool use, various contaminants, including bodily fluids and personal care products, may enter the water. Outdoor pools are additionally exposed to environmental debris such as leaves and rain-borne particles, all of which can introduce pathogenic microorganisms, including bacteria, viruses, and protozoa.^1–3^ Swimming pools are aquatic environments widely used for sports, health, and recreational purposes. To maintain hygienic water quality and prevent the proliferation of waterborne pathogens, disinfection is mandatory, with chlorination being the most common method.^4,5^ The application of chlorination, although essential for water disinfection, promotes chemical interactions with organic matter that result in the formation of disinfection byproducts (DBPs), including trihalomethanes (THMs), haloacetic acids (HAAs), and halogenated acetonitriles (HANs), all of which have been associated with significant health hazards.^6–8^

The formation of DBPs in swimming pool water is driven by precursors originating from both natural and anthropogenic sources, including natural organic matter (NOM) present in the fill water as well as swimmer-derived inputs such as sweat, urine, skin cells, and hair. In addition, personal care products (PCPs) such as sunscreens, lotions, soaps, and shampoos represent a substantial contribution to the overall organic load.^1,9–11^ Due to the typically higher chlorine doses required in pools compared to drinking water, and the elevated water temperatures that promote microbial growth, pool conditions create an environment prone to DBP formation.^12–14^ Persistent chlorine levels of up to 10 mg/L are often maintained in swimming pools, whereas drinking water usually contains less than 2 mg/L.^7,15^ The chemical composition of body fluids further accelerates DBP generation by reacting with chlorine to form THMs, HAAs, and HANs.^16,17^ Although more than 700 DBP species have been described to date,^18,19^ only a limited number have been studied in swimming pool contexts.^1,3,7,15,20^ Among these, nitrogen-containing DBPs (N-DBPs), particularly haloacetonitriles such as bromochloroacetonitrile (BCAN), have gained increasing attention because of their high cytotoxicity and lack of regulatory oversight.^21,22^ Nitrogenous DBP precursors such as urea, ammonia, amino acids, and creatinine, originating from human urine, significantly contribute to their formation.^1^ Although the concentrations of DBPs typically detected in swimming pool water are not acutely toxic, elevated levels have been associated with diverse adverse biological effects, including cytotoxic, genotoxic, mutagenic, carcinogenic, neurotoxic, and teratogenic outcomes.^23–26^

Swimmers may be exposed to DBPs through multiple routes, including dermal contact, inhalation, oral ingestion, buccal absorption, and auricular exposure.^27–29^ Epidemiological studies have shown that DBP exposure in pools often exceeds that from drinking water, posing greater long-term health risks.^30^ While THMs and HAAs are the most frequently monitored, unregulated DBPs such as HANs may be more toxic.^6,31^ The skin, constituting nearly 15% of body weight, acts as a primary defense barrier against environmental insults. During swimming or bathing, skin is directly exposed to DBPs.^32,28,33^ Upon injury, wound healing proceeds through inflammation, proliferation, and remodeling phases.^34^ Incomplete healing increases susceptibility to infection, oxidative stress, and delayed closure.^35^ Reactive oxygen species (ROS), while essential at physiological levels for keratinocyte migration and angiogenesis, become detrimental when present in excess.^36,37^

In vitro models, including HaCaT keratinocytes and HUVEC endothelial cells, are extensively employed to investigate the cellular and molecular impacts of chemical exposures on wound healing, reflecting their respective contributions to re-epithelialization and angiogenesis.^38–41^ HaCaT cells, being immortalized and non-tumorigenic, offer a cost-effective model for assessing cytotoxicity, ROS generation, and cell migration.^42,43^ Recent studies also suggest that exposure to agents such as cold atmospheric plasma (CAP) can modulate these cellular behaviors.^44^ Although DBP toxicity in drinking water has been extensively studied, their dermal effects, particularly those related to wound healing, remain largely unexplored in swimming pool settings. Although the cytotoxicity of disinfection byproducts has been investigated in several cellular systems, studies specifically examining BCAN in skin- and vascular-relevant in vitro models remain limited.^45,46^ In particular, previous studies have mainly focused on general DBP occurrence, cytotoxicity profiling, or oxidative stress responses, whereas data linking BCAN exposure to keratinocyte wound closure and endothelial tube formation are scarce.^45,46^ Therefore, the present study comparatively assessed BCAN-induced changes in viability, membrane integrity, oxidative stress, and regenerative behavior in HaCaT and HUVEC cells, thereby extending the current understanding of the potential dermal and vascular effects of this unregulated N-DBP.^45–47^

## 2 Materials and Methods

### 2.1 Chemicals and Solutions

3-(4,5-Dimethylthiazol-2-yl)-2,5-diphenyltetrazolium bromide (MTT), penicillin–streptomycin solution, fetal bovine serum (FBS), dimethyl sulfoxide (DMSO), endothelial cell growth supplement (ECGS), ethylenediaminetetraacetic acid (EDTA), sodium pyruvate, sodium bicarbonate, and Matrigel basement membrane matrix were purchased from Sigma-Aldrich (St. Louis, Missouri, USA). Ham’s F-12 medium and Dulbecco’s modified Eagle’s medium (DMEM) high glucose were obtained from Capricorn Scientific (Germany). The ROS Detection Assay Kit (K936-250) was purchased from BioVision (Milpitas, California, USA). Cytotoxicity was additionally evaluated using the LDH Cytotoxicity Detection Kit (Roche, Cat. No. 04 744 926 001). Bromochloroacetonitrile (BCAN) was obtained from LGC Dr. Ehrenstorfer (Germany). Methanol used for stock preparation was purchased from Carlo Erba S.A.S.

### 2.2 Preparation of BCAN Concentrations

A 100,000 μM BCAN stock solution was prepared by dissolving 50 mg of BCAN in methanol. A 10,000 μM master stock was then prepared by dilution with sterile distilled water. Working solutions were freshly prepared in culture medium supplemented with 10% FBS, and a 100 μM intermediate solution was used to generate the final treatment concentrations. HaCaT cells were exposed to six BCAN concentrations (5, 10, 20, 40, 60, and 80 μM) for 24 and 48 h. Each concentration was tested in eight replicate wells, and the experiments were repeated independently three times at different time points. Untreated medium controls and solvent controls (methanol at a final concentration of 0.1%, v/v) were evaluated in parallel.

### 2.3 Cell Culture

HaCaT cells were obtained from Cell Line Services (CLS, Eppelheim, Germany), and HUVEC cells were purchased from the American Type Culture Collection (ATCC, Gaithersburg, Maryland, USA). HaCaT cells were cultured in Dulbecco’s Modified Eagle’s Medium supplemented with 10% fetal bovine serum (FBS; Sigma-Aldrich, Germany), 1% penicillin–streptomycin (Sigma-Aldrich, Germany), and 7.5% sodium bicarbonate (NaHCO_3_; AppliChem, Darmstadt, Germany). Cells were maintained in a humidified CO_2_ incubator at 37 °C until reaching approximately 70% confluence.

HUVEC cells were grown in Ham’s F-12 nutrient mixture supplemented with 20% FBS, 1% penicillin–streptomycin, sodium bicarbonate, heparin, and endothelial cell growth supplement (ECGS). Both cell lines were cultured in 25 cm^2^ flasks and incubated at 37 °C in a humidified atmosphere containing 5% CO_2_.

### 2.4 Cell Viability and Cytotoxicity Assays

HaCaT and HUVEC cells were seeded into 96-well plates at densities of 1.0 × 10^4^ and 8.0 × 10^3^ cells/well, respectively, in 100 μL of culture medium containing 10% FBS. After 24 h to allow cell attachment, the cells were treated with the indicated concentrations of BCAN.

The cytotoxic effects of BCAN were evaluated using MTT and LDH assays. The MTT assay was performed as previously described by Mosmann.^48^ After treatment, absorbance was measured at 570 nm using a BioTek 800 TS microplate reader (Winooski, Vermont, USA).

LDH release was measured using the LDH Cytotoxicity Detection Kit (Roche, Germany) according to the manufacturer’s instructions. Following BCAN exposure, culture supernatants were collected, and absorbance was measured at 492 nm using the same microplate reader. LDH release was used as an indicator of cell membrane damage.

Methanol, used as the solvent for BCAN, served as the solvent control. Cell viability was expressed as a percentage relative to the untreated control group, which was defined as 100%. Each BCAN concentration was tested in eight technical replicates, and all experiments were independently repeated three times to ensure reproducibility.

### 2.5 Generation of Reactive Oxygen Species (ROS)

BioVision Kit (K936-250) (Milpitas, California, USA) was used to determine the amount of reactive oxygen species (ROS) in the cell. HaCaT and HUVECs were seeded in a black/clear-bottom 96-well plate (Greiner Bio-One GmbH, Germany) at 1 × 10^4^ and 8 × 10^3^ cells/well. After waiting for 23 h for cells to adhere, the medium was removed. According to the kit procedure, 100 μL of ROS assay buffer was added to each well and the cells were washed. Then, the wells were incubated with 100 μL of 1× ROS marker prepared in ROS assay buffer for 40 min at 37 °C. Then, the 1× ROS marker was removed, freshly prepared BCAN substance 5, 10, 20, 40, 60, and 80 μM concentrations were added to the wells, and the plates were incubated at 37 °C for 24 h. At the end of the incubation period, the change in the amount of ROS in the cell was measured fluorometrically in a SpectraMax M2 microplate reader (Molecular Devices, San Jose, California, USA) at 495/529 nm and the results were compared with the control (untreated group). Methanol, used as the solvent for BCAN, served as the solvent control. Each BCAN concentration was tested in eight technical replicates, and all experiments were independently repeated three times to ensure reproducibility.

### 2.6 Scratch Wound Healing Assay

In the scratch wound healing assay, HaCaT cells (5 × 10^5^) were seeded in six-well cell culture plates (TPP, Techno Plastic Products AG, Germany) and allowed to grow to 70–80% confluence as a monolayer. The resulting monolayer was gently drawn in a straight line from the center of each well with a sterile 200 μL pipet tip. After drawing, the medium was removed and the wells were washed in PBS (Sigma) solution, BCAN substance doses prepared with DMEM containing 10% fetal bovine serum, 1% penicillin–streptomycin, and 7.5% NaHCO_3_ were added to each well, and cells were added to 24 and grown in a 48 h CO_2_ incubator. Scratch wound healing of images was observed under an Olympus IX71 inverted microscope (Tokyo, Japan) and photographed with an Olympus DP70 camera (Tokyo, Japan) at 10× magnification at 0, 24, and 48 h after scratch wound formation in HaCaT cells. Experiments were carried out in three replicates.^49^

### 2.7 Endothelial Tube Formation Assay

The endothelial tube formation assay was conducted following established protocols.^49^ HUVECs were serum-starved by incubating them in EBM-2 medium supplemented with 2% FBS for 4 h. Subsequently, 4 × 10^4^ HUVECs per well were seeded onto Matrigel-coated 96-well plates and exposed to EBM-2 medium containing the test compounds. After 12 h of incubation, tube formation was visualized using an Olympus IX71 inverted microscope (Tokyo, Japan) and documented with an Olympus DP70 camera (Tokyo, Japan) at 4× magnification. Experiments were carried out in three replicates.

### 2.8 Statistical Evaluation

Statistical analyses were performed using SPSS version 21.0 (IBM Corp., Armonk, New York, USA). Data obtained from MTT, LDH, and ROS assays are presented as mean ± standard deviation (SD) of at least three independent experiments, each conducted with multiple technical replicates. All experiments included appropriate controls, including untreated cells and solvent controls. For quantitative assays (MTT, LDH, and ROS), each experimental condition was performed in eight technical replicates (*n* = 8), whereas tube formation assays were conducted in triplicate (*n* = 3). All experiments were independently repeated at least three times to ensure reproducibility and reliability. Data were normalized to the control group, which was set at 100% viability. The normality of data distribution was assessed using the Shapiro–Wilk test. Statistical comparisons were performed using one-way analysis of variance (ANOVA) followed by Tukey’s post hoc multiple comparison test. Differences were considered statistically significant at *p* < 0.05, and significant differences from the control group are indicated by asterisks in the figures.

## 3 Results

### 3.1 Effect of BCAN on Cell Viability in HaCaT and HUVEC Cells

The cytotoxic effects of BCAN were evaluated in HaCaT and HUVEC cells using the MTT assay after 24 and 48 h treatments with concentrations ranging from 5 to 80 μM. In HaCaT cells, a time- and dose-dependent decrease in cell viability was observed. After 24 h, viability was reduced to 97% (5 μM), 88% (10 μM), 85–86% (20 μM), 84% (40 μM), 70% (60 μM), and 65% (80 μM), compared to the control. After 48 h, viability decreased further to 83% (5 μM), 75–76% (10 μM), 64% (20 μM), 50% (40 μM), 44% (60 μM), and 42–43% (80 μM), indicating a significant cytotoxic response in HaCaT cells (Figure 1A). Based on these data, the half-maximal inhibitory concentration (IC_5__0_) of BCAN in HaCaT cells at 48 h was calculated to be approximately 42 μM. In contrast, BCAN did not significantly affect the viability of HUVEC cells at concentrations of 5–60 μM, with viability values comparable to the control group at both time points. At 80 μM, only a slight reduction of approximately 6% was observed after 48 h (Figure 1B). These findings suggest that BCAN selectively induces cytotoxicity in HaCaT cells in a dose- and time-dependent manner, while HUVEC cells remain largely unaffected under the same treatment conditions.

**Figure 1:**
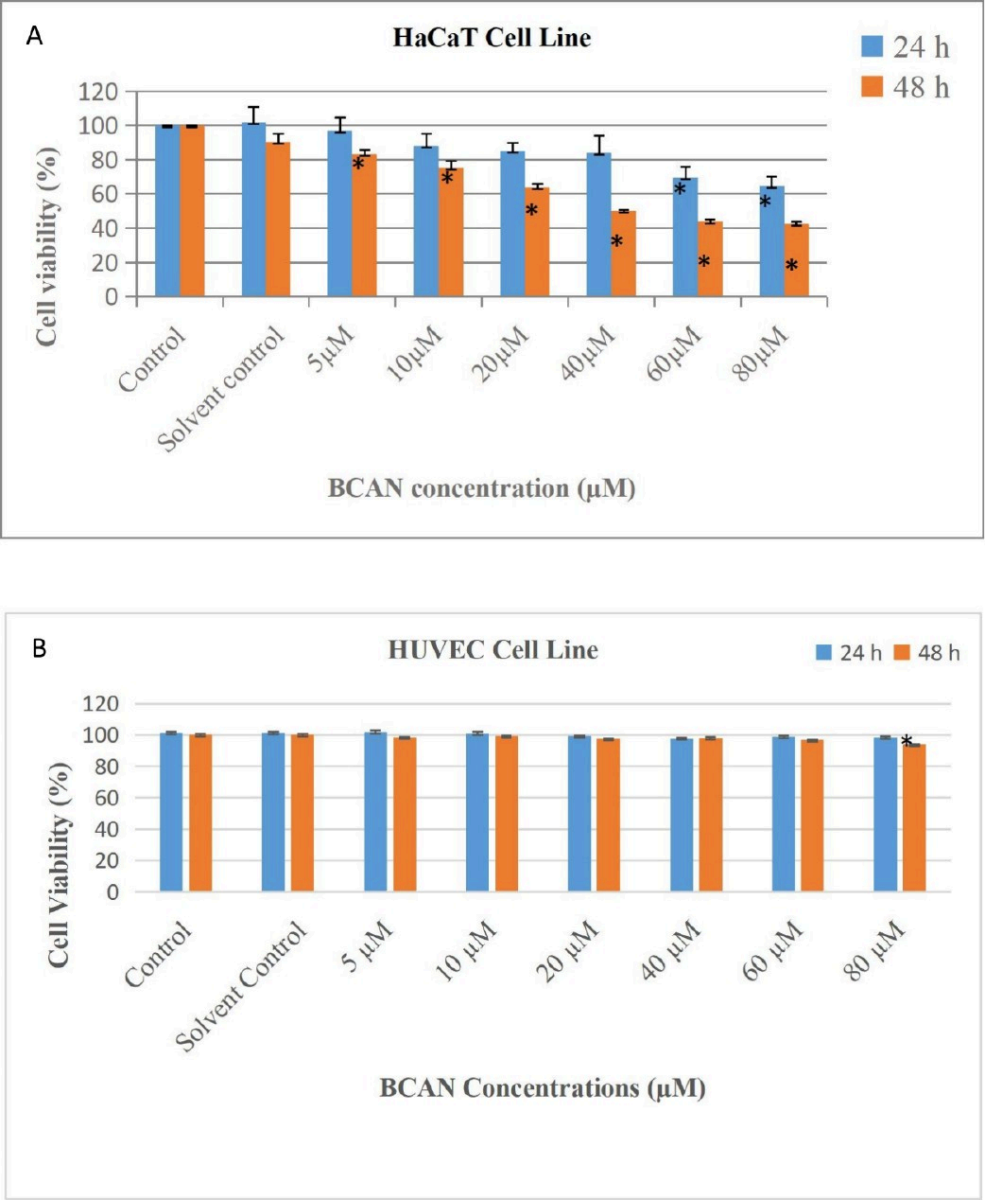
Evaluation of BCAN-induced cytotoxicity in HaCaT (A) and HUVEC (B) cells at 24 and 48 h by MTT assay. Cells were treated with BCAN at concentrations of 5, 10, 20, 40, 60, and 80 μM. Statistically significant differences compared to the control group are indicated by an asterisk (*), with significance defined as *p* < 0.05.

### 3.2 LDH Release in HaCaT Cells Following BCAN Treatment

Membrane integrity and cell damage were evaluated by measuring LDH release in HaCaT and HUVEC cells following 24 and 48 h treatments with BCAN at concentrations ranging from 5 to 80 μM. In HaCaT cells, LDH release increased in a concentration-dependent manner after 24 h, with elevations of 3% (5 μM), ∼5% (10 μM), 8–9% (20 μM), 11% (40 μM), 12% (60 μM), and 13% (80 μM) compared to the control. After 48 h, LDH release increased further to 5% (5 μM), ∼16% (10 μM), 23% (20 μM), 26% (40 μM), 27% (60 μM), and ∼29% (80 μM), demonstrating a clear time- and dose- dependent cytotoxic effect in HaCaT cells (Figure 2A). In contrast, HUVEC cells showed no significant changes in LDH release at concentrations of 5–60 μM across both time points. Only at 80 μM was a moderate increase of approximately 9% observed after 48 h (Figure 2B). Combined data from both the MTT and LDH assays indicate that BCAN-induced cytotoxicity and membrane damage occur selectively in HaCaT cells under the tested conditions.

**Figure 2:**
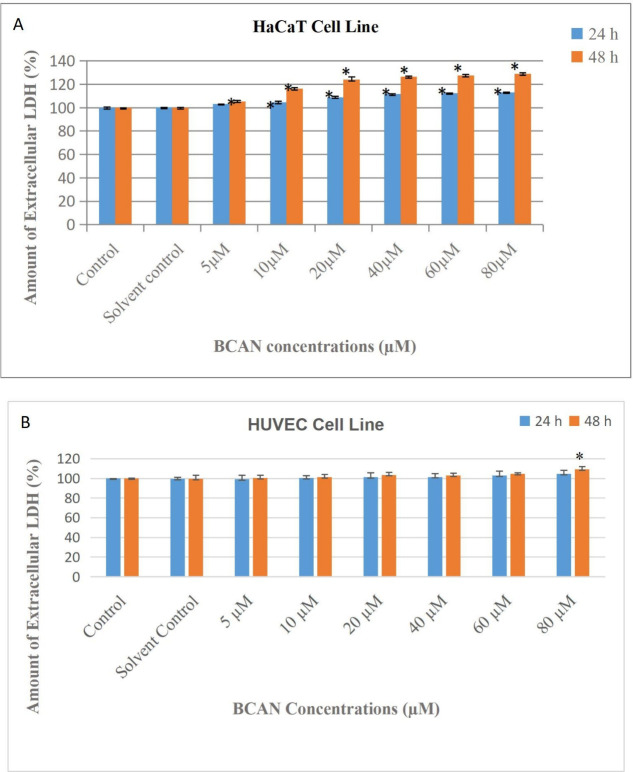
Assessment of BCAN-induced cytotoxicity in HaCaT (A) and HUVEC (B) cells following 24 and 48 h of treatment by LDH assay. Cells were exposed to BCAN at concentrations of 5, 10, 20, 40, 60, and 80 μM. Statistically significant differences relative to the control group are denoted by an asterisk (*), with significance set at *p* < 0.05.

### 3.3 Intracellular ROS Generation in HaCaT and HUVEC Cells Following BCAN Exposure

The intracellular reactive oxygen species (ROS) levels were measured in HaCaT and HUVEC cells after 24 h exposure to BCAN at concentrations of 5, 10, 20, 40, 60, and 80 μM. In HaCaT cells, BCAN treatment led to a concentration-dependent increase in ROS production compared to the control. Specifically, ROS levels increased by 6–7% (5 μM), 7–8% (10 μM), 8–9% (20 μM), 10% (40 μM), 18–19% (60 μM), and 32–33% (80 μM), indicating enhanced oxidative stress at higher concentrations (Figure 3A).

**Figure 3:**
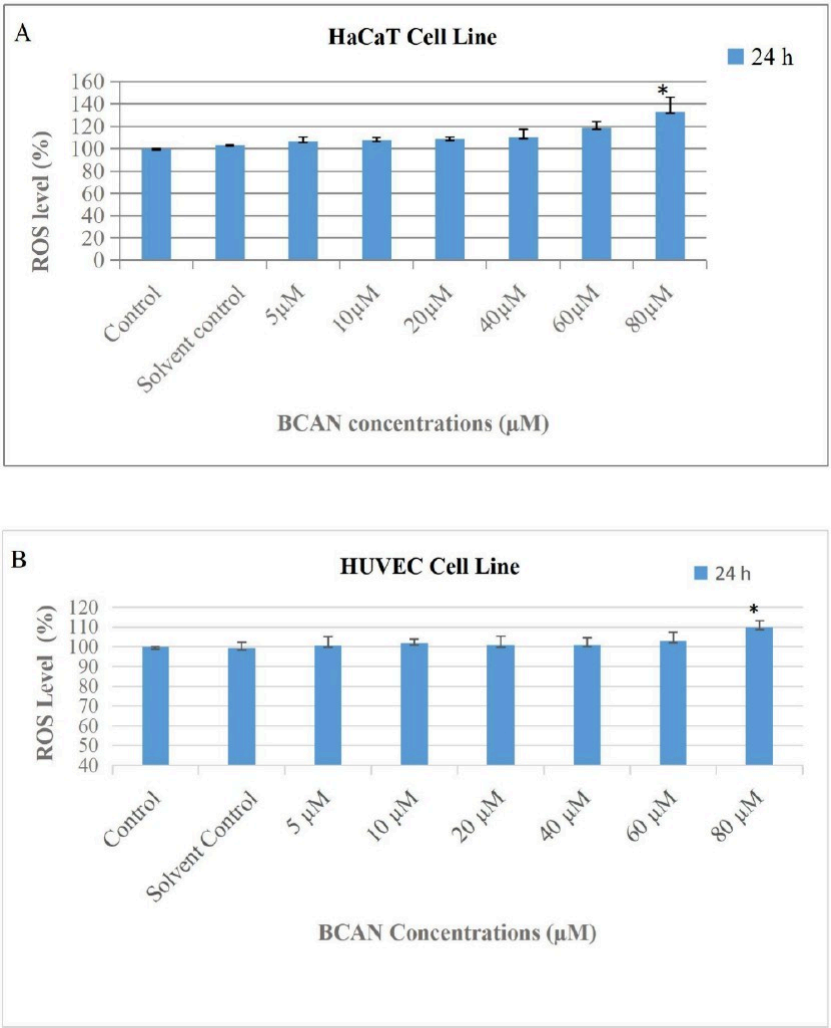
Evaluation of BCAN-induced reactive oxygen species (ROS) production in HaCaT (A) and HUVEC (B) cells following 24 h of treatment. Cells were exposed to BCAN at concentrations of 5, 10, 20, 40, 60, and 80 μM. Statistically significant differences compared to the control group are indicated by an asterisk (*), with significance defined as *p* < 0.05.

In contrast, HUVEC cells showed no significant changes in ROS levels at concentrations up to 60 μM. A statistically significant increase (∼9%) was observed only at the highest concentration (80 μM), suggesting that BCAN-induced oxidative stress is more prominent in HaCaT cells under the tested conditions (Figure 3B).

### 3.4 Scratch Assay Findings

The biological activity of different concentrations of BCAN was tested in an *in vitro* wound healing model using HaCaT cells. Scratch wound healing of images obtained using an Inverted IX71 (Olympus) microscope and DP70 camera at 0, 24, and 48 h after scratch wound formation in HaCaT cells of 5, 10, 20, 40, 60, 80 μM BCAN concentrations were evaluated.

Wound healing was observed after 24 h in both the control and solvent control groups. BCAN treatment at noncytotoxic concentrations (5, 10, and 20 μM) did not visibly impair keratinocyte migration or wound closure compared with the control groups under the present experimental conditions. A slight qualitative increase in wound closure appeared to be present at 10 and 20 μM; however, this observation was not quantitatively assessed.

At higher concentrations (40, 60, and 80 μM), which are associated with increased oxidative and cytotoxic stress, only a moderate delay in wound closure was observed. However, keratinocyte migration was still detectable, and wound closure was not entirely inhibited. This suggests that despite the oxidative burden at these higher concentrations, HaCaT cells retained a degree of regenerative capacity and migration potential (Figure 4).

**Figure 4:**
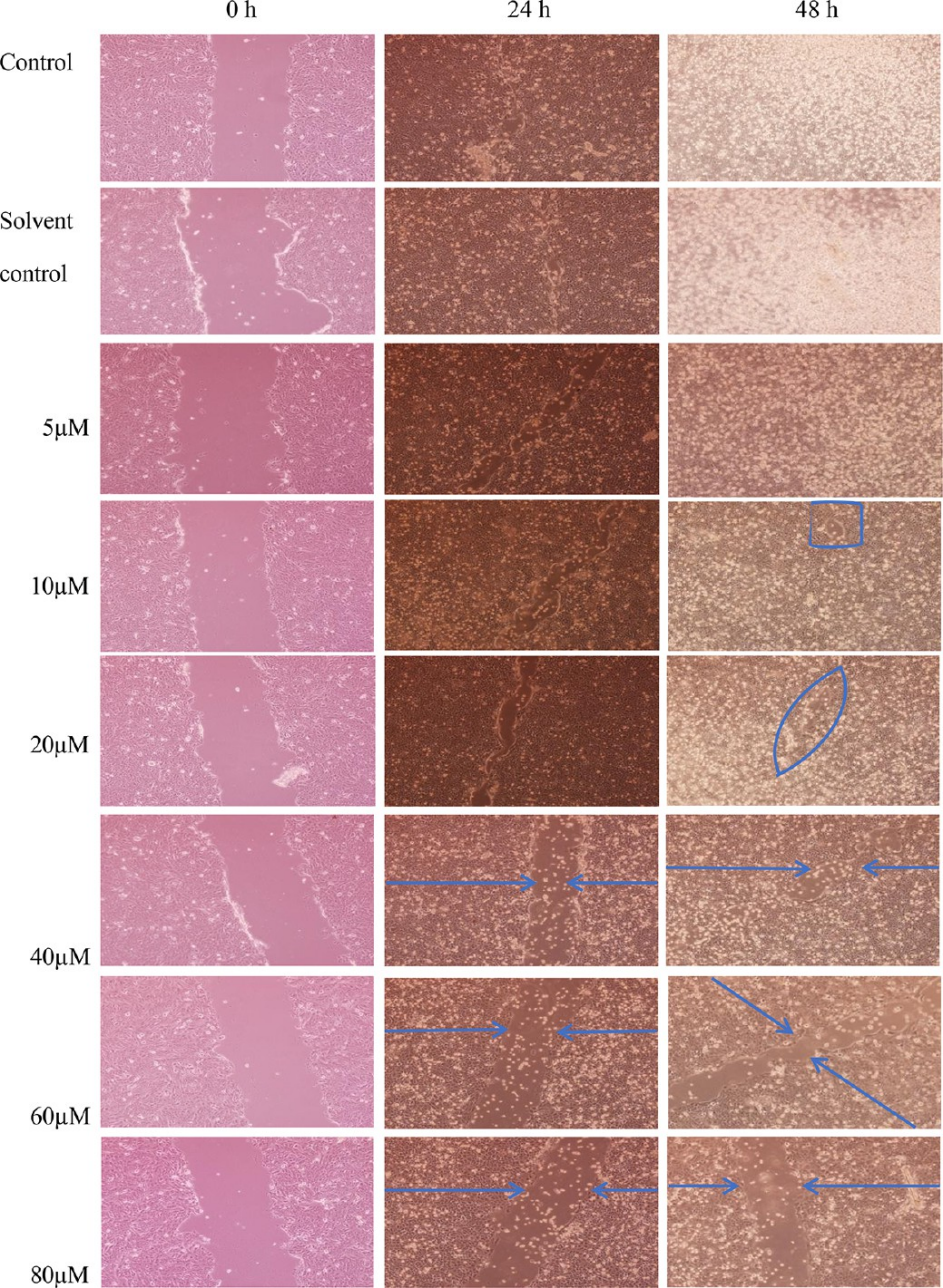
In vitro wound healing activity of BCAN at various concentrations in HaCaT cells at 0 (left), 24 (middle), and 48 (right) hours after scratch. Wound closure was assessed in control, solvent control, and BCAN-treated groups at concentrations of 5, 10, 20, 40, 60, and 80 μM.

The qualitative scratch assay findings suggest that BCAN may not markedly impair keratinocyte-mediated wound closure under the present experimental conditions, although this interpretation would benefit from quantitative image analysis.

### 3.5 Endothelial Tube Formation Assay

To evaluate the angiogenic potential of BCAN, an endothelial tube formation assay was conducted following 12 h of treatment. In both the control and vehicle groups, HUVEC cells successfully formed well-organized, interconnected capillary-like tubular structures. Similarly, at all tested concentrations of BCAN, the integrity of tube and network formation was largely preserved, and no significant reduction in cell number or structure formation was observed. The representative tube formation images did not reveal an obvious inhibitory effect of BCAN on angiogenic network formation under the present experimental conditions; however, quantitative angiogenesis metrics would be needed to confirm this observation (Figure 5).

**Figure 5:**
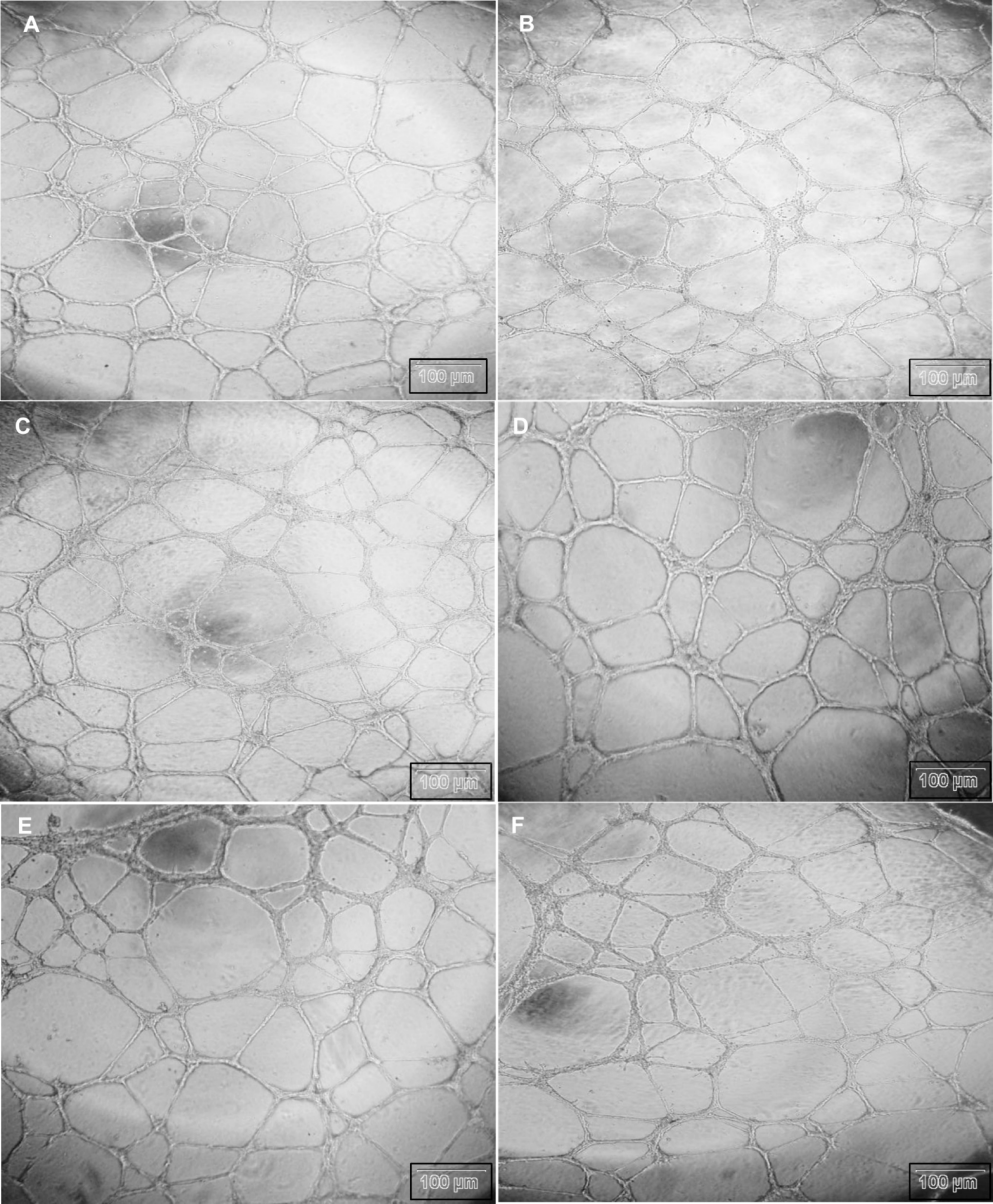
Effects of BCAN on endothelial tube formation in HUVECs. Representative images of (A) control cells, (B) solvent control cells, and HUVECs treated with BCAN at (C) 20, (D) 40, (E) 60, and (F) 80 μM. Images correspond to independent triplicate experiments. Scale bar: 100 μm.

## 4 Discussion

This study provides new evidence on the cytotoxic and regenerative effects of bromochloroacetonitrile (BCAN), a nitrogenous disinfection byproduct (N-DBP), in human skin keratinocytes (HaCaT) and vascular endothelial cells (HUVEC). As chlorination byproducts are frequently detected in swimming pools and have been associated with various adverse health outcomes, understanding their dermal and vascular impacts is critical. For instance, eye, respiratory, and skin irritations in swimmers have been clearly linked to exposure to DBPs such as trichloramine, whereas evidence for long-term outcomes (e.g., asthma or cancer) from pool use remains inconclusive.^50,51^ Notably, disinfection byproduct (DBP) concentrations in swimming pool waters often exceed those found in drinking water due to continuous organic matter input and persistent disinfectant presence. For example, one survey found that swimming pools contained total DBP concentrations nearly two orders of magnitude higher than their fill water supplies, and trihalomethane levels in some pools have been measured in the hundreds of μg/L far exceeding those typically found in drinking water.,^29,3^ However, the effects of pool operational parameters on DBP formation and speciation have not been systematically characterized, complicating toxicological safety assessments of pool water.^17^ Moreover, swimming pool DBPs have attracted comparatively less scientific and public attention than those in drinking water.^13^

Research has predominantly focused on trihalomethanes (THMs), haloacetic acids (HAAs), and chloramines especially trichloramine while nitrogenous DBPs such as haloacetonitriles (HANs) and haloketones (HKs) remain underexplored. This is of concern, as nitrogenous DBPs like HANs tend to exhibit greater genotoxicity and cytotoxicity than the more commonly regulated carbonaceous DBPs. Moreover, regulated THMs themselves contribute only a minor fraction of total cytotoxicity; high THM levels mainly serve as indicators of more toxic DBPs (e.g., HANs) rather than direct risk drivers.^6,47,52^ Because carbonaceous and nitrogenous DBPs coexist and nitrogenous DBP formation may increase when THM and HAA production is suppressed, simultaneous investigation of both groups during disinfection is essential. Consistent with recent findings, although nitrogenous DBPs account for only 6–18% of total DBP species, they contribute disproportionately up to 70% to overall DBP toxicity, with BCAN identified as one of the most critical contributors.^22^

Swimming is considered one of the most accessible and beneficial forms of physical activity worldwide, appealing to people of all ages and socioeconomic groups.^2,50,53^ However, the realization of these health benefits depends on maintaining well-controlled aquatic environments. During use, pools may become contaminated through bodily secretions, cosmetic residues, and environmental inputs such as rain or wind-blown debris, particularly in outdoor settings. These inputs can introduce microbial pathogens including bacteria, viruses, and protozoa that may lead to infections like gastroenteritis or dermatitis.^3,54^ As such, maintaining effective disinfection strategies is vital to minimize health risks associated with recreational water use.^1^

While numerous DBPs have been investigated in the context of drinking water and swimming pool toxicity, most studies have emphasized occurrence patterns, general cytotoxicity ranking, or oxidative stress-related end points rather than regenerative cell behavior.^45,46^ In this context, the present study extends the available literature by integrating cytotoxicity, membrane damage, oxidative stress, and regenerative readouts in two biologically relevant cell models. Rather than focusing solely on viability, this work comparatively evaluates HaCaT and HUVEC responses to BCAN and includes functional end points related to keratinocyte wound closure and endothelial tube formation. This broader design may help refine the biological interpretation of BCAN toxicity beyond conventional acute cytotoxicity end points.^45,46^

Our results demonstrated that HaCaT keratinocytes exhibited clear dose- and time-dependent sensitivity to BCAN. Metabolic activity (MTT assay) was significantly impaired at concentrations ≥20 μM, with an IC_50_ of ∼42 μM after 48 h. These findings are consistent with previous studies highlighting the potent cytotoxicity of haloacetonitriles (HANs), including oxidative stress–linked toxicity and apoptosis-related responses.,^55,56^ In contrast, HUVEC cells maintained viability and membrane integrity across the same concentration range, with only mild impairment observed at the highest concentration (80 μM), suggesting a cell-type-specific sensitivity to BCAN. Such differences may reflect variations in antioxidant capacity, membrane permeability, or cellular uptake mechanisms between keratinocytes and endothelial cells.

Oxidative stress emerged as a key mechanism underlying BCAN-induced toxicity. In HaCaT cells, BCAN exposure led to a concentration-dependent increase in intracellular reactive oxygen species (ROS), peaking at 80 μM with a 32–33% increase compared to control. ROS accumulation was modest in HUVECs and only reached statistical significance at the highest concentration tested. These findings suggest that oxidative stress may contribute to BCAN-induced cytotoxicity, potentially involving NRF2-linked protective signaling.^55,57^ The literature indicates that NRF2 activation and redox homeostasis serve as protective responses to ROS insults, and their upregulation may help explain the resilience observed in HUVECs.^57^

Interestingly, despite pronounced oxidative and cytotoxic stress in HaCaT cells at higher BCAN doses, keratinocyte migration and wound closure capacity were not markedly impaired. Keratinocyte migration is central to re-epithelialization and effective wound closure.^58^ Our results demonstrated that BCAN at subtoxic concentrations (5–20 μM) did not impair wound healing in HaCaT cells, with only modest delays observed at higher doses (40–80 μM). Notably, these concentrations exceed cytotoxic thresholds reported for commonly used mammalian models (e.g., CHO cells) exposed to HANs.^11,14,59,60^ Likewise, acute cytotoxicity in hepatoma models has been reported for several HANs, with effects linked to oxidative stress and DNA damage, broadly consistent with the oxidative stress profile observed here.^56,21,61^

One plausible explanation for the apparent dissociation between oxidative stress and impaired wound closure is the context-dependent role of ROS in keratinocyte biology. Moderate ROS levels can promote migration and remodeling, whereas excess ROS disrupts cytoskeletal dynamics and signaling.^34,62^ Our data suggest that HaCaT cells retain functional resilience in wound repair, even under oxidative challenge, possibly through adaptive cytoprotective responses. Likewise, moderate intracellular ROS can act as secondary messengers to promote cell adhesion and migration via redox-regulated pathways, whereas excessive ROS leads to molecular damage and cell death.^63,64^

Similarly, HUVEC angiogenic capacity remained largely preserved across BCAN concentrations, with tube formation assays showing maintained capillary-like network formation at 20–60 μM and only mild suppression at 80 μM, in parallel with reduced viability. To the best of our knowledge, studies directly examining the effects of BCAN on endothelial tube formation remain limited, and the present findings provide initial evidence in this area.

From an environmental health perspective, the relevance of these findings must be interpreted in the context of real-world exposure levels. BCAN concentrations in swimming pools are typically detected in the low μg/L range, several hundred times lower than the lowest concentration (5 μM ≈ 800 μg/L) tested in this study.^30,3^ For instance, BCAN levels of 2.8–20.8 μg/L have been reported in chlorinated pool water, reinforcing that typical environmental exposures are far below our experimental doses.^30,3^ Although the concentrations examined here exceeded reported environmental levels, they were deliberately selected within a literature-supported in vitro range relevant for haloacetonitrile toxicity studies, allowing direct comparison with prior cytotoxicity data while, for the first time, extending assessment to wound healing and endothelial tube formation end points.^55,56^ However, concerns remain regarding chronic low-dose exposure, particularly in frequent swimmers or sensitive populations, which warrants further investigation.

In summary, BCAN exerts selective cytotoxic and oxidative effects in human skin keratinocytes, while endothelial cells display a higher degree of functional and biochemical resilience. Importantly, regenerative functions such as wound closure and angiogenesis were not substantially inhibited at any tested dose, underscoring the functional tolerance of these cell types to short-term BCAN exposure. These findings enhance our toxicological understanding of emerging N-DBPs and emphasize the need to evaluate both cytotoxicity and regenerative capacity in environmental risk assessments.

From a public health perspective, this study highlights the importance of minimizing nitrogenous DBP formation in swimming pools through effective water quality management. Future research should prioritize long-term, low-dose exposure models (e.g., 3D human skin equivalents and in vivo systems) to assess the potential for cumulative dermal toxicity or impaired tissue regeneration.^65^ Studies exploring the combined effects of multiple DBPs and their impact on inflammatory signaling, DNA damage, or cell cycle regulation would deepen mechanistic insights. Additionally, investigating protective strategies such as antioxidant supplementation or enhancing NRF2-mediated defenses could help mitigate risks in highly exposed populations.^55,57^ These efforts will inform regulatory guidelines and support the development of integrated disinfection approaches including improved preswim hygiene and alternative technologies (e.g., UV, ozone) to reduce DBP formation and ensure safer recreational water environments.^1,66^

## 5 Conclusions

This study provides evidence that bromochloroacetonitrile (BCAN), a highly cytotoxic nitrogenous disinfection byproduct (N-DBP), exerts selective oxidative and metabolic toxicity in human keratinocytes (HaCaT), while endothelial cells (HUVEC) remain comparatively resistant. Despite elevated ROS levels and membrane damage at concentrations ≥40 μM, HaCaT cells retained significant wound closure capacity, suggesting that adaptive cytoprotective pathways may help preserve functional regenerative responses under stress that preserve functional regenerative responses under stress. Notably, angiogenic behavior in HUVECs appeared to be preserved at all but the highest concentrations, reinforcing the notion of cell-type-specific sensitivity.

The partial dissociation between cytotoxicity and regenerative function observed here highlights the complexity of DBP-induced cellular responses and underscores the necessity of evaluating not only cell viability but also functional outcomes when assessing dermal risk. While the concentrations used in this study exceed environmentally reported levels, they enabled the characterization of threshold mechanisms relevant to acute exposures.

From a regulatory and public health standpoint, these findings underscore a critical knowledge gap in the toxicological profiling of N-DBPs, particularly in the context of dermal exposure during recreational water use. To date, most studies have focused on acute, high-dose exposure models. However, the data presented herein strongly suggest that future investigations should emphasize chronic, low-dose exposure paradigms that better reflect real-life scenarios. Taken together, these findings may support future efforts to refine long-term risk assessment frameworks for DBPs in swimming pool water, particularly with respect to low-concentration and chronic exposure scenarios.

## Data Availability

All data generated or analyzed during this study are included in this published article.
